# Supplementary material for: Cognitive computational model reveals repetition bias in a sequential decision-making task
Source: Commun Psychol. 2025 Jun 13;3:92. doi: 10.1038/s44271-025-00271-0 (PMC12166051; doi:10.1038/s44271-025-00271-0)
Supplement: Supplementary file 1 — Supplementary Information [file 44271_2025_271_MOESM1_ESM.pdf]

Cognitive Computational Model Reveals Repetition Bias in a Sequential Decision-Making Task—Supplementary Information

Eric Legler[1], Darío Cuevas Rivera[1,2], Sarah Schwöbel[1], Ben J. Wagner[1], and Stefan Kiebel[1,2]

[1]Chair of Cognitive Computational Neuroscience, Faculty of Psychology, TUD Dresden University of Technology, Dresden, Germany
[2]Centre for Tactile Internet with Human-in-the-Loop (CeTI), TUD Dresden University of Technology, Dresden, Germany

Contents

1 Supplementary Figures **2**

2 Supplementary Tables **7**

1 Supplementary Figures

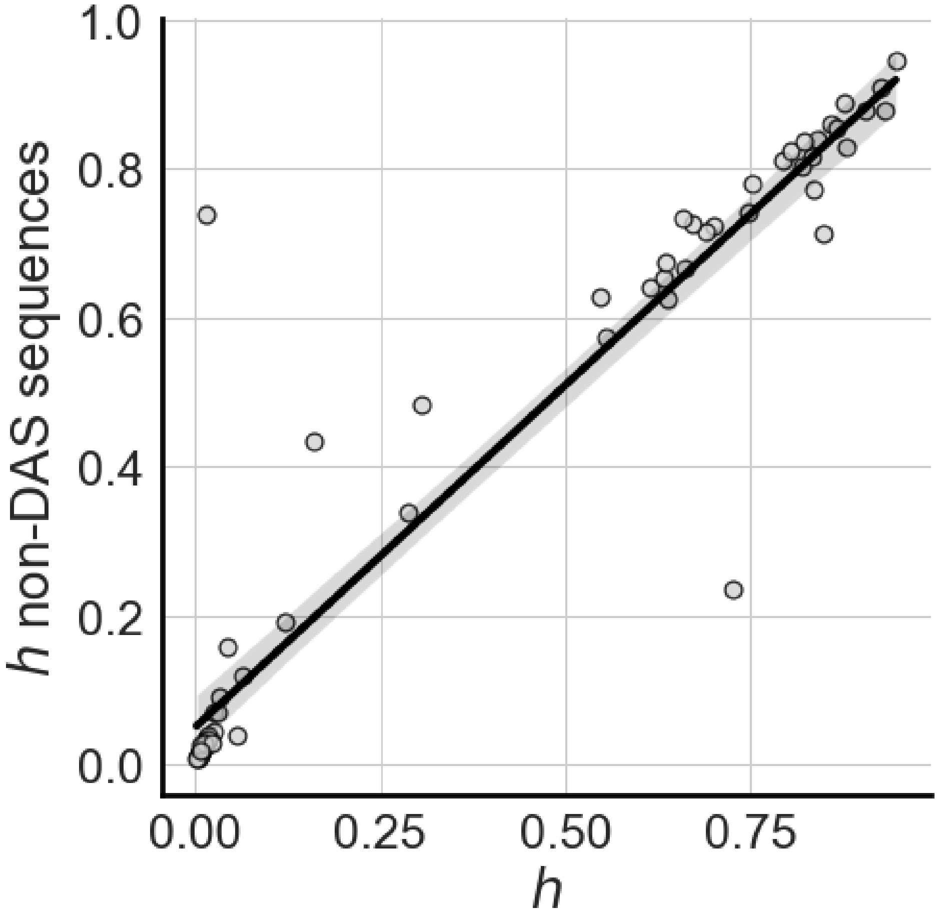

Supplementary Figure 1: **Correlation between general repetition bias strength h of EVPRM and non-DAS h of alternative EVPRM model.** Correlation between the repetition bias strength parameter h of EVPRM that represents a general repetition bias over all sequences, and the h parameter for all non-DAS sequences of the alternative EVPRM model, that has separate h parameters for DAS and all non-DAS sequences. Black solid line represents linear regression model fitted to the data. Gray area represents 95% confidence interval for the regression. $N = 70$ participants.

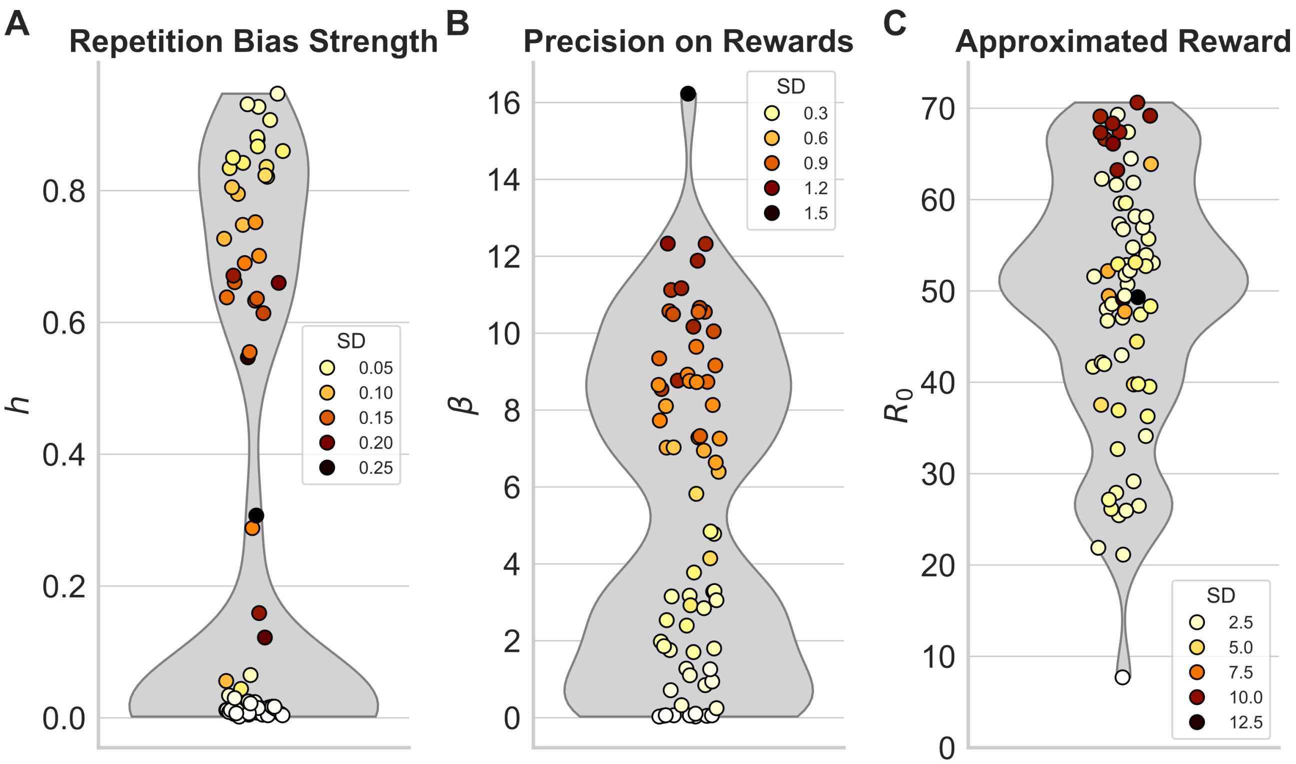

Supplementary Figure 2: **Estimated parameters of expected value with proxy and repetition bias model (EVPRM)** Dots represent posterior means of individual parameter estimates. Each plot represents one of the three free parameters: **(A)** repetition bias strength h, **(B)** precision on expected rewards β and **(C)** approximated reward R_0. Gray patches represent kernel density estimates. The color of dots indicate standard deviations (SD). $N = 70$ participants.

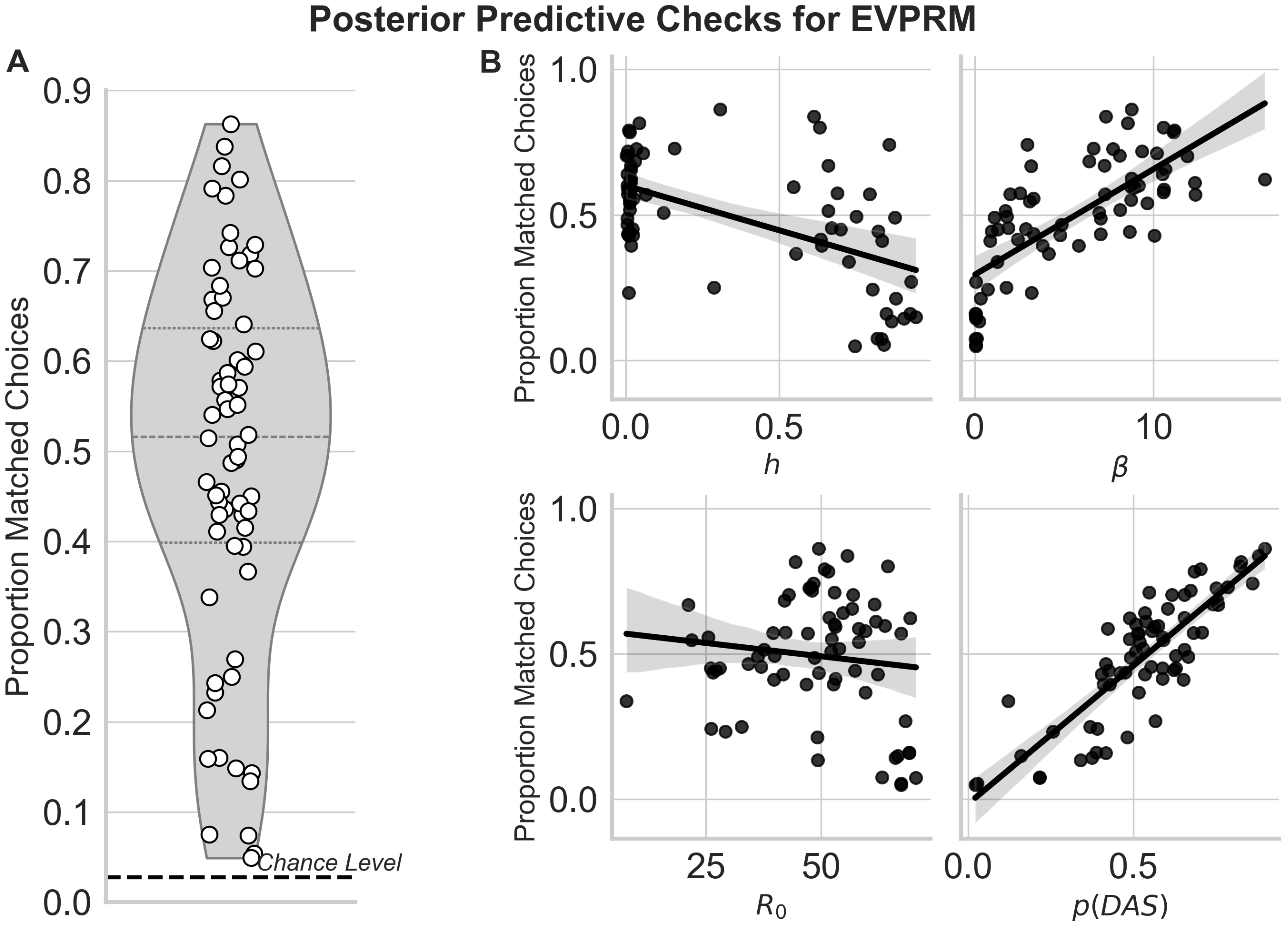

Supplementary Figure 3: **Posterior predictive checks (PPC) for expected value with proxy and repetition bias model (EVPRM).** **(A)** Distribution of the proportion correctly predicted choices for each participant based on simulated data with the inferred parameters from the EVPRM. Each white dot represents the proportion of correctly predicted choices for one participant. The gray area represents a kernel density estimate (KDE) of the distribution, and the dotted and dashed lines inside the KDE represent the borders of the quartiles. Dashed line represents chance level. **(B)** Correlations between proportion of correctly predicted choices of each participant and the three inferred parameters of the EVPRM and their proportion of default action sequence (DAS) choices. Black solid lines represent linear regression model fitted to the data. h: repetition bias strength, β: precision over expected rewards, R_0: approximated reward, $p(\text{DAS})$: proportion of DAS choices. Gray areas represent 95% confidence interval for the regression. $N = 70$ participants.

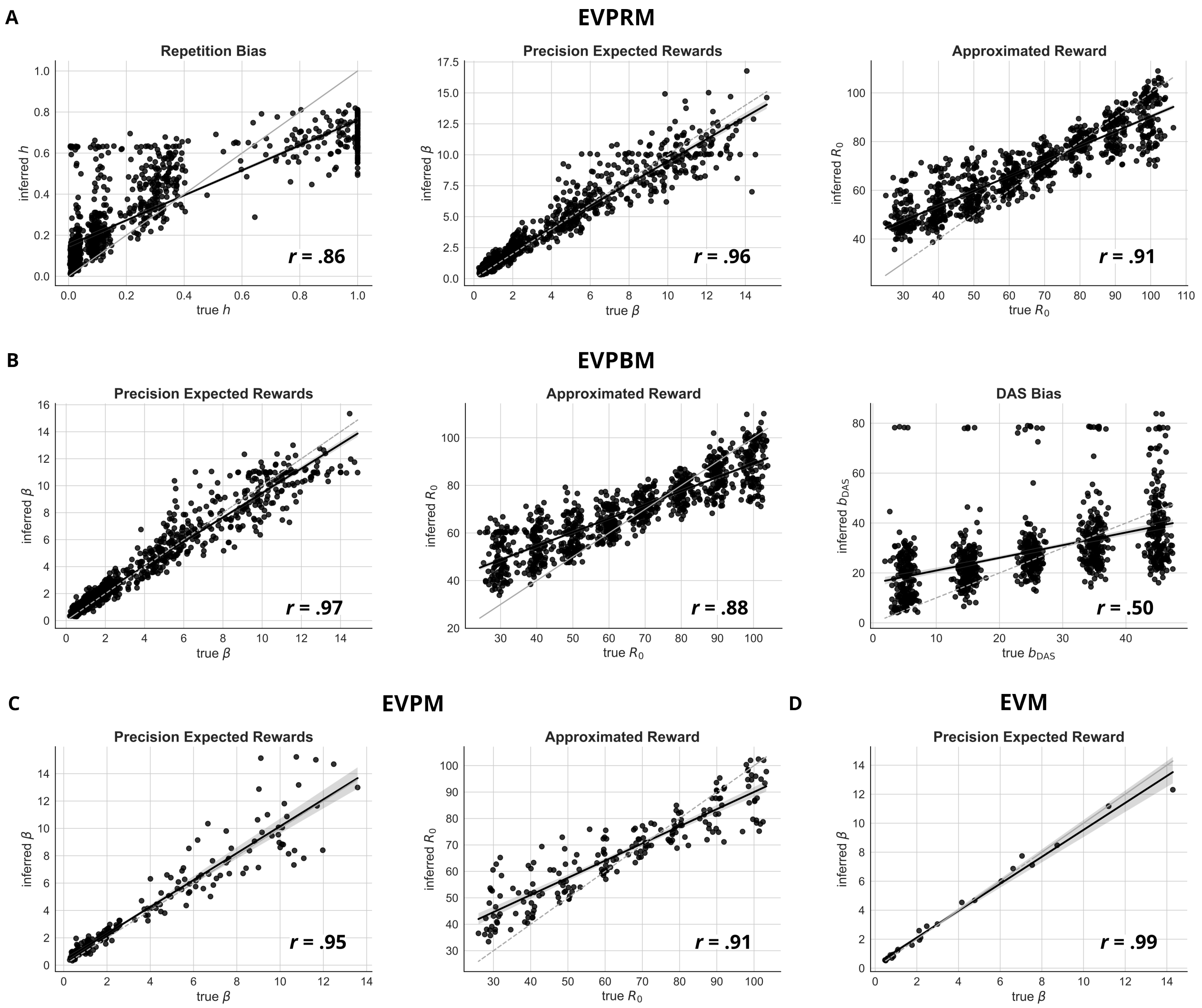

Supplementary Figure 4: **Parameter recovery for all candidate models.** Correlations of true and inferred parameter values for all free parameters of the four candidate models: (**A**) expected value with proxy and repetition bias model (EVPRM), (**B**) expected value with proxy and default bias model (EVPBM) (**C**) expected value with proxy model (EVPM), (**D**) and expected value model (EVM). Black solid lines represent correlation between true and inferred parameter values. Grey dashed lines represent true parameter values.

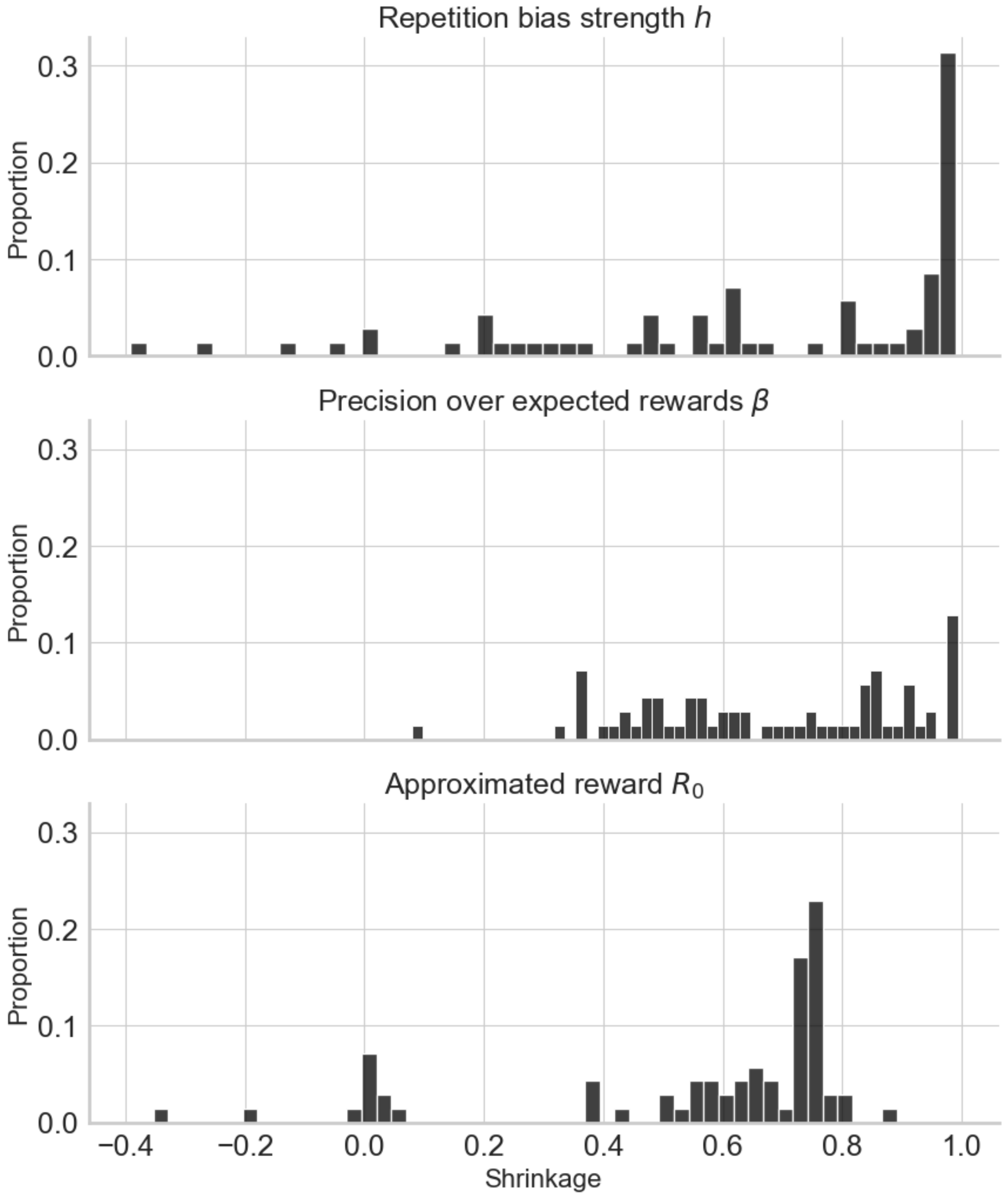

Supplementary Figure 5: **Shrinkage between prior and posterior for EVPRM.** Distribution of shrinkage for model parameters of EVPRM based on the prior and posterior standard deviations for each parameter. $N = 70$ participants.

2 Supplementary Tables

Supplementary Table 1: Descriptive statistics depending on bonus condition

	All Trials		Bonus		No Bonus		*t*-Test			
	M	*SD*	*M*	*SD*	*M*	*SD*	*t*	*p*	*d*	*CI*
p(DAS)	0.57	0.15	0.57	0.19	0.52	0.19	-4.68	<.001	.26	[-∞, -0.03]
Reward	81.52	5.00	81.34	6.63	80.04	5.92	-2.84	.003	.21	[-∞, -0.54]
RT (*ms*)	1,645.17	335.21	1,691.79	405.11	1,662.76	407.69	-1.56	.938	.07	[-60.16, ∞]
Time Outs	4.70	3.63	2.60	2.49	2.14	1.91	-1.56	.938	.21	[-0.01, ∞]

Depicted are means for all trials, trials with potential bonus for DAS, and trials without potential bonus for DAS over all participants. The *t*-tests represent one-sided *t*-tests for related samples that test for significant differences between bonus and no bonus trials. For *p*(DAS) and reward we tested if the means of the first half are smaller than the means of the second half. For RT and time outs we tested if the means of the first half are greater than the means of the second half. *p*(DAS): proportion of default action sequence (DAS) choices, reward: mean reward per trial, RT: reaction time, *M*: mean, *SD*: standard deviation, *t*: *t*-statistic, *p*: *p*-value, *d*: Cohen's *d*, *CI*: 95% confidence intervals of the difference in means.

Supplementary Table 2: Correlations between inferred parameter values of EVPRM and performance measures

	h	β	R_0	p(DAS)	Reward	RT
β	-0.75	-				
R_0	**0.12**	**0.30**	-			
p(DAS)	-0.22	**0.37**	-0.18	-		
Reward	**-0.69**	**0.76**	-0.11	**0.67**	-	
RT	**0.37**	**-0.44**	**0.25**	**-0.58**	**-0.70**	-
Time outs	**0.32**	**-0.27**	0.16	**-0.40**	**-0.52**	**0.65**

Depicted are correlation coefficients between inferred parameter values of EVPRM and performance measures. As performance measures the means of each participant were used. Significant correlations are bold ($p < .05$). β: precision over expected rewards, R_0: approximated reward, h: repetition bias strength, p(DAS): proportion of DAS choices, reward: mean reward per trial, RT: reaction time, DAS: default action sequence, EVPRM: expected value with proxy and repetition bias model.

Supplementary Table 3: Summary of posterior distributions of EVPRM

	h			β			R_0		
ID	M	SD	95% HDI	M	SD	95% HDI	M	SD	95% HDI
0	0.01	0.01	[0.00, 0.03]	10.56	0.93	[8.90, 12.50]	59.58	2.28	[55.01, 63.82]
1	0.03	0.02	[0.01, 0.08]	7.73	0.79	[6.25, 9.32]	47.42	3.54	[40.41, 54.22]
2	0.93	0.04	[0.85, 0.99]	0.03	0.01	[0.01, 0.06]	69.18	9.86	[49.34, 87.96]
3	0.02	0.03	[0.00, 0.03]	3.15	0.26	[2.65, 3.67]	21.14	2.61	[16.10, 26.03]
4	0.01	0.01	[0.00, 0.03]	11.12	1.03	[9.03, 13.12]	51.58	2.48	[46.65, 56.32]
5	0.02	0.01	[0.00, 0.03]	16.23	1.60	[13.17, 19.31]	69.29	2.00	[65.50, 73.25]
6	0.12	0.21	[0.00, 0.66]	6.95	0.65	[5.70, 8.23]	52.18	6.03	[43.70, 65.42]
7	0.06	0.04	[0.01, 0.14]	12.33	1.16	[10.09, 14.59]	67.40	2.58	[62.59, 72.54]
8	0.95	0.03	[0.89, 0.99]	0.05	0.01	[0.03, 0.07]	66.65	9.35	[48.56, 84.62]
9	0.01	0.00	[0.00, 0.02]	3.28	0.27	[2.76, 3.82]	26.48	2.71	[21.42, 31.96]
10	0.84	0.07	[0.70, 0.97]	0.06	0.02	[0.02, 0.10]	67.39	9.69	[49.77, 86.83]
11	0.29	0.13	[0.05, 0.53]	1.76	0.22	[1.36, 2.22]	32.69	3.58	[25.66, 39.69]
12	0.04	0.07	[0.00, 0.15]	8.55	1.02	[6.62, 10.57]	44.45	4.61	[35.73, 54.34]
13	0.31	0.26	[0.01, 0.79]	8.77	1.10	[6.59, 10.90]	49.45	6.12	[38.76, 59.74]
14	0.55	0.24	[0.00, 0.85]	8.73	0.87	[7.06, 10.50]	63.89	5.65	[48.00, 69.98]
15	0.70	0.12	[0.48, 0.94]	1.28	0.15	[1.00, 1.58]	27.90	3.33	[21.95, 34.91]
16	0.93	0.04	[0.86, 0.99]	0.06	0.02	[0.02, 0.10]	68.33	9.87	[50.16, 88.14]
17	0.02	0.01	[0.00, 0.03]	12.32	1.09	[10.29, 14.51]	61.85	2.23	[57.30, 66.04]
18	0.88	0.06	[0.75, 0.98]	1.10	0.17	[0.79, 1.44]	36.27	4.08	[28.68, 44.45]
19	0.80	0.10	[0.60, 0.97]	1.98	0.27	[1.47, 2.52]	39.52	3.97	[32.07, 47.51]
20	0.01	0.00	[0.00, 0.01]	9.34	0.88	[7.65, 11.08]	48.03	2.49	[43.11, 52.77]
21	0.84	0.08	[0.66, 0.97]	0.85	0.16	[0.53, 1.16]	39.77	4.82	[30.27, 49.22]
22	0.01	0.00	[0.00, 0.02]	3.17	0.26	[2.67, 3.70]	29.15	2.61	[24.13, 34.18]
23	0.75	0.10	[0.56, 0.94]	0.05	0.02	[0.01, 0.10]	67.32	9.81	[48.29, 86.60]
24	0.00	0.00	[0.00, 0.01]	7.02	0.65	[5.80, 8.29]	48.57	2.79	[43.15, 53.80]
25	0.01	0.00	[0.00, 0.01]	7.29	0.63	[6.02, 8.49]	47.08	2.65	[42.09, 52.42]
26	0.75	0.12	[0.52, 0.96]	1.80	0.26	[1.31, 2.30]	39.80	4.04	[31.67, 47.65]
27	0.01	0.00	[0.00, 0.01]	10.57	0.93	[8.74, 12.39]	58.17	2.31	[53.66, 62.72]
28	0.02	0.01	[0.00, 0.05]	4.78	0.43	[3.98, 5.66]	41.70	3.09	[35.97, 48.16]
29	0.88	0.06	[0.76, 0.98]	0.32	0.18	[0.04, 0.64]	49.12	11.91	[29.21, 72.42]
30	0.02	0.02	[0.01, 0.05]	3.29	0.29	[2.74, 3.88]	25.45	2.88	[19.93, 30.97]
31	0.66	0.15	[0.38, 0.93]	7.26	0.74	[5.87, 8.80]	61.62	2.52	[56.61, 66.33]
32	0.80	0.10	[0.62, 0.96]	0.72	0.10	[0.52, 0.92]	26.14	3.76	[19.12, 33.54]
33	0.00	0.00	[0.00, 0.01]	9.16	0.86	[7.55, 10.92]	52.84	2.56	[47.94, 57.80]
34	0.00	0.00	[0.00, 0.01]	10.49	0.95	[8.59, 12.32]	54.77	2.39	[50.04, 59.32]
35	0.01	0.01	[0.00, 0.02]	8.65	0.76	[7.16, 10.16]	57.31	2.42	[52.28, 61.78]
36	0.01	0.01	[0.00, 0.02]	8.92	0.78	[7.46, 10.48]	53.07	2.46	[47.99, 57.55]
37	0.01	0.00	[0.00, 0.02]	8.13	0.75	[6.67, 9.64]	53.92	2.78	[48.55, 59.57]
38	0.69	0.13	[0.43, 0.92]	2.54	0.35	[1.90, 3.22]	42.18	3.90	[33.93, 49.34]
39	0.02	0.01	[0.00, 0.03]	10.66	0.97	[8.85, 12.63]	56.74	2.44	[51.77, 61.26]
40	0.83	0.08	[0.68, 0.96]	0.03	0.01	[0.00, 0.06]	70.62	9.77	[52.86, 90.61]
41	0.67	0.18	[0.33, 0.97]	1.86	0.29	[1.32, 2.41]	36.94	4.42	[28.01, 45.69]
42	0.01	0.01	[0.00, 0.02]	11.17	1.10	[9.13, 13.46]	50.72	2.49	[45.53, 55.40]
43	0.06	0.10	[0.00, 0.24]	10.17	1.09	[8.15, 12.41]	52.92	4.30	[44.93, 62.40]
44	0.64	0.14	[0.38, 0.92]	3.78	0.43	[2.98, 4.66]	52.72	3.52	[45.70, 59.41]
45	0.01	0.01	[0.00, 0.03]	3.06	0.24	[2.61, 3.53]	21.89	2.40	[17.01, 26.30]
46	0.82	0.08	[0.66, 0.97]	0.10	0.04	[0.03, 0.19]	63.23	9.84	[43.62, 82.58]
47	0.82	0.08	[0.66, 0.98]	0.94	0.12	[0.71, 1.19]	27.16	3.48	[20.68, 34.31]
48	0.00	0.00	[0.00, 0.01]	4.84	0.39	[4.06, 5.56]	34.12	2.59	[28.74, 38.85]
49	0.85	0.07	[0.71, 0.98]	0.06	0.02	[0.02, 0.11]	69.10	9.51	[51.29, 88.41]
50	0.00	0.00	[0.00, 0.00]	8.10	0.69	[6.75, 9.42]	42.97	2.33	[38.16, 47.27]
51	0.02	0.01	[0.00, 0.03]	8.76	0.78	[7.37, 10.40]	51.77	2.58	[46.76, 56.71]
52	0.02	0.01	[0.00, 0.03]	5.82	0.51	[4.89, 6.86]	46.74	2.79	[41.22, 52.08]
53	0.66	0.20	[0.20, 0.98]	1.71	0.32	[1.15, 2.36]	37.53	4.94	[27.68, 47.30]
54	0.01	0.01	[0.00, 0.02]	8.73	0.77	[7.28, 10.27]	52.23	2.51	[46.98, 56.79]

55	0.61	0.16	[0.30, 0.91]	7.32	0.90	[5.61, 9.17]	55.70	3.06	[49.63, 61.53]
56	0.86	0.08	[0.71, 0.98]	2.92	0.47	[2.03, 3.86]	48.32	4.06	[40.59, 56.29]
57	0.63	0.15	[0.35, 0.91]	10.56	0.88	[8.80, 12.27]	64.48	2.00	[60.30, 68.15]
58	0.01	0.00	[0.00, 0.02]	10.05	0.96	[8.19, 11.90]	62.26	2.46	[57.58, 67.13]
59	0.91	0.05	[0.81, 0.99]	0.06	0.03	[0.01, 0.12]	66.11	10.17	[47.08, 86.50]
60	0.87	0.07	[0.74, 0.98]	0.25	0.15	[0.04, 0.56]	49.31	13.39	[25.44, 73.12]
61	0.64	0.15	[0.35, 0.91]	2.40	0.39	[1.65, 3.16]	53.12	4.48	[43.94, 61.51]
62	0.56	0.14	[0.28, 0.83]	4.14	0.52	[3.12, 5.18]	59.63	3.31	[53.31, 66.12]
63	0.01	0.00	[0.00, 0.02]	11.89	1.12	[9.83, 14.14]	56.94	2.31	[52.44, 61.41]
64	0.73	0.10	[0.53, 0.92]	1.26	0.08	[1.11, 1.42]	7.70	1.07	[5.67, 9.80]
65	0.01	0.01	[0.00, 0.02]	9.65	0.83	[7.92, 11.23]	58.12	2.43	[53.29, 62.84]
66	0.03	0.02	[0.01, 0.06]	6.40	0.62	[5.17, 7.61]	41.98	3.12	[35.80, 47.86]
67	0.16	0.19	[0.01, 0.60]	6.64	0.69	[5.31, 7.97]	47.74	6.21	[38.39, 60.74]
68	0.02	0.01	[0.01, 0.04]	2.85	0.25	[2.38, 3.36]	25.95	2.67	[20.70, 31.04]
69	0.01	0.00	[0.00, 0.02]	7.03	0.57	[5.85, 8.09]	49.49	2.42	[44.52, 53.93]

Posterior distributions for all participants for EVPRM. h: repetition bias strength, β: precision on expected rewards, R_0: approximated reward, M: mean, SD: standard deviation, 95% HDI: 95% highest density interval.
