# Supplementary material for: Cognitive computational model reveals repetition bias in a sequential decision-making task
Source: Commun Psychol. 2025 Jun 13;3:92. doi: 10.1038/s44271-025-00271-0 (PMC12166051; doi:10.1038/s44271-025-00271-0)
Supplement: Supplementary file 2 — Reporting Summary [file 44271_2025_271_MOESM2_ESM.pdf]

nature portfolio

Corresponding author(s): Eric Legler
Last updated by author(s): May 2, 2025

Reporting Summary

Nature Portfolio wishes to improve the reproducibility of the work that we publish. This form provides structure for consistency and transparency in reporting. For further information on Nature Portfolio policies, see our Editorial Policies and the Editorial Policy Checklist.

Statistics

For all statistical analyses, confirm that the following items are present in the figure legend, table legend, main text, or Methods section.

n/a	Confirmed	
☐	☒	The exact sample size (*n*) for each experimental group/condition, given as a discrete number and unit of measurement
☐	☒	A statement on whether measurements were taken from distinct samples or whether the same sample was measured repeatedly
☐	☒	The statistical test(s) used AND whether they are one- or two-sided<br>*Only common tests should be described solely by name; describe more complex techniques in the Methods section.*
☒	☐	A description of all covariates tested
☐	☒	A description of any assumptions or corrections, such as tests of normality and adjustment for multiple comparisons
☐	☒	A full description of the statistical parameters including central tendency (e.g. means) or other basic estimates (e.g. regression coefficient) AND variation (e.g. standard deviation) or associated estimates of uncertainty (e.g. confidence intervals)
☐	☒	For null hypothesis testing, the test statistic (e.g. *F*, *t*, *r*) with confidence intervals, effect sizes, degrees of freedom and *P* value noted<br>*Give P values as exact values whenever suitable.*
☐	☒	For Bayesian analysis, information on the choice of priors and Markov chain Monte Carlo settings
☒	☐	For hierarchical and complex designs, identification of the appropriate level for tests and full reporting of outcomes
☐	☒	Estimates of effect sizes (e.g. Cohen's *d*, Pearson's *r*), indicating how they were calculated

Our web collection on statistics for biologists contains articles on many of the points above.

Software and code

Policy information about availability of computer code

Data collection	Experimental task was coded with lab.js (https://labjs.felixhenninger.com/) and hosted on the neurotests server (https://neurotests.zih.tu-dresden.de/) from the TUD Dresden University of Technology.
Data analysis	All analyses were performed with Python using the packages numpy, pandas, ArviZ, pingouin, PyMC, and Scipy's stats module. Plots were created with seaborn and matplotlib.

For manuscripts utilizing custom algorithms or software that are central to the research but not yet described in published literature, software must be made available to editors and reviewers. We strongly encourage code deposition in a community repository (e.g. GitHub). See the Nature Portfolio guidelines for submitting code & software for further information.

Data

Policy information about availability of data

All manuscripts must include a data availability statement. This statement should provide the following information, where applicable:

- Accession codes, unique identifiers, or web links for publicly available datasets
- A description of any restrictions on data availability
- For clinical datasets or third party data, please ensure that the statement adheres to our policy

The data for this study are available at OSF https://doi.org/10.17605/OSF.IO/RH42A

Research involving human participants, their data, or biological material

Policy information about studies with human participants or human data. See also policy information about sex, gender (identity/presentation), and sexual orientation and race, ethnicity and racism.

Reporting on sex and gender	Participants were asked to self-report their gender. Our findings do not apply to only one gender.
Reporting on race, ethnicity, or other socially relevant groupings	We did not collect participants' race, ethnicity, or other socially relevant groupings.
Population characteristics	See above.
Recruitment	Participants were recruited using the recruitment system of the faculty of psychology at the TUD Dresden University of Technology. In this system, students and individuals from the general population interested in being participants in psychological studies can register. Only participants with no dyschromatopsia were allowed to take part in the study.
Ethics oversight	The study was approved by the Institutional Review Board of the TUD Dresden University of Technology (protocol number EK 578122019).

Note that full information on the approval of the study protocol must also be provided in the manuscript.

Field-specific reporting

Please select the one below that is the best fit for your research. If you are not sure, read the appropriate sections before making your selection.

☐ Life sciences ☒ Behavioural & social sciences ☐ Ecological, evolutionary & environmental sciences

For a reference copy of the document with all sections, see nature.com/documents/nr-reporting-summary-flat.pdf

Behavioural & social sciences study design

All studies must disclose on these points even when the disclosure is negative.

Study description	All participants (N=74) performed a sequential decision-making task online. After an extensive training phase, participants had to 16 blocks with 20 trials each. Participants had a maximum of 6s to execute the four move sequence to finish each trial. All responses and reaction times were collected.
Research sample	50 out of 70 participants were female, and 20 male. Participants had a mean age of 24.1 years (SD=4.6).
Sampling strategy	We did not perform sample size calculations for our model-free analyses prior to data collection, as the combination of the task with the computational model was not previously used, and therefore we could not estimate expected effect sizes from existing literature. We used parameter recovery and posterior predictive checks to validate our model-based analyses.
Data collection	Data collection was performed online. Participants had to complete informed consent and demographic data at RedCAP. Following complete input of data, participants received a link to the neurotests platform (https://neurotests.zih.tu-dresden.de), where the experimental task was hosted. Only PCs were allowed to perform the task (i.e. no tablets or smartphones).
Timing	Data was collected between and 08.09.2021 and 10.11.2021. There was no gap.
Data exclusions	We excluded 4 participants due to lack of behavioral variability.
Non-participation	52 participants completed the informed consent but did not start to perform the experimental task or did not finish the task.
Randomization	Participants were not allocated into experimental groups.

Reporting for specific materials, systems and methods

We require information from authors about some types of materials, experimental systems and methods used in many studies. Here, indicate whether each material, system or method listed is relevant to your study. If you are not sure if a list item applies to your research, read the appropriate section before selecting a response.

Materials & experimental systems

n/a	Involved in the study
☒	☐ Antibodies
☒	☐ Eukaryotic cell lines
☒	☐ Palaeontology and archaeology
☒	☐ Animals and other organisms
☒	☐ Clinical data
☒	☐ Dual use research of concern
☒	☐ Plants

Methods

n/a	Involved in the study
☒	☐ ChIP-seq
☒	☐ Flow cytometry
☒	☐ MRI-based neuroimaging

Plants

Seed stocks	*Report on the source of all seed stocks or other plant material used. If applicable, state the seed stock centre and catalogue number. If plant specimens were collected from the field, describe the collection location, date and sampling procedures.*
Novel plant genotypes	*Describe the methods by which all novel plant genotypes were produced. This includes those generated by transgenic approaches, gene editing, chemical/radiation-based mutagenesis and hybridization. For transgenic lines, describe the transformation method, the number of independent lines analyzed and the generation upon which experiments were performed. For gene-edited lines, describe the editor used, the endogenous sequence targeted for editing, the targeting guide RNA sequence (if applicable) and how the editor was applied.*
Authentication	*Describe any authentication procedures for each seed stock used or novel genotype generated. Describe any experiments used to assess the effect of a mutation and, where applicable, how potential secondary effects (e.g. second site T-DNA insertions, mosiacism, off-target gene editing) were examined.*
